# *Rickettsia rickettsii* and *Rickettsia typhi* in hospitalized children diagnosed with Pediatric Multisystemic Inflammatory Syndrome in Merida, Yucatan

**DOI:** 10.1016/j.bjid.2023.103707

**Published:** 2023-12-06

**Authors:** Karla Dzul-Rosado, Dayana Guadalupe Lavín-Sánchez, Gerardo Álvarez Hernández, Salvador Gómez-Carro, Fernando I. Puerto-Manzano

**Affiliations:** a Hideyo Noguchi Regional Investigation Center, Faculty of Medicine, Autonomous University of Yucatan, Mérida, México; b Department of Medicine and Health Sciences, University of Sonora, Sonora, México; c Agustín O' Horan General Hospital, Department of Health, Mérida, Yucatán, México

**Keywords:** Rickettsiosis, MIS-C, Fever, Inflammatory markers

## Abstract

Multisystem Inflammatory Syndrome in Children (MIS-C) presents with fever, fatigue, elevated inflammatory markers (acute phase reactants), and a history of exposure to SARS-CoV-2 or positive antibodies to SARS-CoV-2. As the COVID-19 pandemic unfolded, the risk of MIS-C in the pediatric population increased. However, exposure to other viruses and the presence of SARS-CoV-2 positive antibodies in children hospitalized for various pathogen-associated illnesses will also remain common and may complicate differential diagnoses with diseases endemic to the region such as rickettsial diseases. The objective was to highlight the desirability of medical personnel systematically incorporating rickettsiosis as a differential diagnosis for MIS-C when studying a child with fever, non-specific symptoms, and elevated inflammatory markers. In conclusion MIS-C should be considered in children with elevated inflammatory markers when there is a history of COVID-19 and they also meet criteria that have already been established by international agencies, such as CDC and WHO

## Introduction

Multisystem Inflammatory Syndrome in Children (MIS-C) presents with fever, fatigue, elevated inflammatory markers and a recent history of COVID-19 infection or the presence of positive antibodies to SARS-CoV-2.^1^ It is linked to multiorgan involvement, including rapid progression of myocardial dysfunction requiring inotropic support.^2^ The case definition proposed by the U.S. Centers for Disease Control and Prevention (CDC) is highly sensitive in identifying all suspected cases of MIS-C.^3^ However, it has been noted that this definition lacks sufficient specificity and may lead to confusion in differentiating diagnoses.

In Yucatan, two medical conditions that can complicate diagnosis of MIS-C are Typhus Group (TG) and Spotted Fever Group (SFG) Rickettsiosis, mainly in hospitalized children with severe clinical manifestations^4^^,^^5^ Rickettsial diseases are zoonoses caused by bacteria of the genus Rickettsia, which target blood and endothelial cells. They are transmitted by hematophagous arthropods such as ticks, fleas, and lice, which use wild and domestic mammals as reservoirs, while humans are accidental hosts. In addition to delaying the diagnosis of other treatable illnesses, concerns about MIS-C can lead to overuse of health care services, including testing and transfers to tertiary care facilitie.^6^

The onset of the clinical picture of rickettsiosis is nonspecific, with symptoms such as headache, fever, loss of appetite, myalgia, and irritability. Between 40 % and 70 % of patients may develop a centripetal maculopapular rash on the 3rd‒4th day of evolution, which in late stages of the disease may become petechial and purpuric. In severe cases, petechiae can form ecchymotic plaques and produce tissue necrosis; it also causes systemic symptoms including myocarditis, acute renal failure, and vascular collapse, in addition to effects on the central nervous system often resulting in meningismus, psychomotor disturbances, seizures, ataxia, coma or hearing loss.^7^

Once the COVID-19 health emergency has subsided, it is very likely that the disease will adopt endemic behavior that may lead to medical complications such as MIS-C in the pediatric population. In this scenario, MIS-C may bias the clinical suspicion of rickettsiosis in regions where the latter is endemic. We document two children hospitalized for suspected MIS-C who ultimately received positive diagnoses of murine typhoid and spotted fever due to *Rickettsia rickettsii*. Our aim is that health care providers routinely incorporate rickettsiosis as a differential diagnosis for MIS-C when studying a pediatric patient with fever, nonspecific symptoms, and elevated inflammatory markers.

## Case study 1

A 7-year-old female patient from a rural community near Merida, with no history of living with animals, although she had a recent history of tick bite on two occasions. Three weeks earlier she showed symptoms suggestive of a flu-like illness that affected the family, but no tests for SARS-CoV-2 virus were performed.

Five days before admission to the hospital, she presented with unquantified fever, epigastric pain, insomnia, headache, dizziness, and pain in the extremities. She received medical treatment with amoxicillin, dosage unknown, but there was no improvement. She was referred to hospital for unquantified fever > 3 days, exanthema, gastrointestinal symptoms, and elevated inflammatory markers such as D-dimer 7.58 mg/dL (0‒0.55 mg/dL), c-reactive protein 12.59 mg/dL (0‒0.75 mg/dL), procalcitonin 7.03 ng/mL (< 0.5 ng/mL), ferritin 750 ng/mL (11‒306.8 ng/mL), and an IgG + antibody test for SARS-CoV-2, all of which meets the criteria for MIS-C according to the WHO.

On admission to the hospital, she was found to be neurologically intact, with a saburral tongue covered in red spots, pale mucous membranes, evidence of dehydration and erythematous macular rash on the chest and abdomen. Her temperature was 39.5 °C and it was decided to start her on immunoglobulin 1 g/kg/dose due to laboratory data of systemic inflammatory response. Due to the patient's background and current clinical condition, a diagnosis of rickettsiosis was established, and support was requested from the Dr. Hideyo Noguchi Regional Research Center (RRC), while the patient was started on doxycycline (4 mg/kg/day). Fragments of the region of the *ompB* gene (and of the *htrA* gene (17 kDa protein) were amplified and were 98 % homologous to *Rickettsia typhi*.^8^

During her first 24 h of hospitalization, she experienced febrile peaks of up to 38.0 °C and petechial lesions in the lower extremities, as well as pharyngeal hyperemia. Over the following three days, a single circular erythematous lesion appeared in the right groin, resembling an insect bite. The fever continued with peaks at 38.0 °C or less. Details on the laboratory biomarkers are shown in Table 1. On the 4th day, clinical improvement, platelet increase, and quantitative decrease of inflammatory markers were observed. After 72 h without febrile peaks, the patient was discharged due to improvement, with the addition of four days treatment with doxycycline (50 mg every 24 h for 12 days).

**Table 1:** Laboratory follow-up during hospital stay, with focus on the first day on which empirical antibiotic treatment was started (case study 1).

Variables	Day 1	Day 4	Day 7	Reference values
	(19/07)	(22/07)	(25/07)	
**Hematic Biometry**				
Leukocytes (mm^3^)	7.4	6.5	9.2	4.5‒10 mm^3^
Neutrophils (%)	79.5	54.90	30.6	37 %‒73 %
Hemoglobin (g/dL)	13.8	12	12.1	10‒15 g/dL
Hematocrit (g/dL)	39.5	34.9	36.1	30‒46 g/dL
Platelets (mm³)	78	146	311	150‒400 mm^3^
**Chemistry**				
Total Bilirubin (mg/dL)	0.5	0.3	0.3	0.3‒1 mg/dL
Direct Bilrrubin (mg/dL)	0.1	0.0	0.0	0‒0.20 mg/dL
Indirect Bilirubina (mg/dL)	0.4	0.3	0.3	
AST (UI/L)	49	35	93	15‒41 UI/L
ALT (U/L)	67	49	79	15‒41 UI/L
Lactate Dehydrogenase (U/L)	472	405.77	366.7	140‒271 UI/L
**Inflammatory Markers**				
D-Dimer (mg/dL)	7.58	0.85	No data	0‒0.55 mg/dL
Ferritin (ng/mL)	750.4	499.8	No data	11‒306.8 ng/mL
Procalcitonin (ng/mL)	7.03	No data	No data	< 0.05 ng/mL
CRP (mg/dL)	12.59	4.89	0.94	0‒0.75 mg/dL

CRP, C-Reactive Protein; ALT, Alanine aminotransferase or serum glutamate-pyruvate transaminase; AST, Aspartate Transaminase or Serum glutamic oxaloacetic transaminase.

## Case study 2

A 6-year-old male, treated at a private clinic for generalized petechial rash and pruritus. His condition started on August 12, with fever up to 38.5°, mitigated with paracetamol. Three days later, generalized reddish macular exanthema appeared. After eight days, he presented asthenia, adynamia, intense generalized pruritus, and ataxic gait, and was therefore transferred to the nearest medical facility, where he was admitted with somnolence, stupor, dehydration, generalized maculopapular exanthema, hyperreflexia, and increased cutaneous sensitivity, the diagnosis being Pediatric Multisystemic Inflammatory Syndrome. It was decided to treat the patient with immunoglobulin. In order for him to have access to specialized medical care, he was sent to a second level hospital.

On arrival at the hospital, he was found to have severe thrombocytopenia of 18,000 mm^3^ and therefore three platelet concentrates were transfused; he also had elevated D-dimer 16.94 mg/dL (0‒0.55 mg/dL), fibrinogen 201.4 mg/dL (200‒500 mg/dL), Alkaline Phosphatase and Lactate Dehydrogenase, as well as mild hypokalemia and hypocalcemia. He had antibodies to SARS-CoV- 2 IgG (+), IgM (-) (Table 2). A suspected diagnosis of Kawasaki phenotype multisystemic inflammatory response syndrome was established, and he was consequently treated with human immunoglobulin at 2 g/kg divided into 2 doses. After 12 h of hospital stay, he showed neurological deterioration and tonic seizures of 15 min duration, and he was admitted to the Pediatric Emergency Room (PER).

**Table 2:** Laboratory results on admission to hospital (case study 2).

Variables	Count	Reference values
**Hematic Biometry**		
Leukocytes (mm^3^)	19.50	4.5‒10 mm^3^
Neutrophils (%)	78.20	37 %‒73 %
Hemoglobin (g/dL)	11.90	10‒15 g/dL
Hematocrit (g/dL)	35	30‒46 g/dL
Platelets (mm^3^)	18	150‒400 mm^3^
**Chemistry**		
Glucose	87	70‒105 mg/dL
BUN	23.4	7‒25 mg/dL
Urea	50.1	17.10‒42.80 mg/dL
Creatinin	0.6	0.70‒1.30 mg/dL
Uric Acid	3.3	2.50‒7 mg/dL
**Inflammatory Markers**		
D-Dimer (mg/dL)	16.94	0‒0.55 mg/dL
Procalcitonin (ng/mL)	5.29	< 0.05 ng/mL
CRP (mg/dL)	15.04	0‒0.75 mg/dl

During his stay in the PER he underwent a cranial CT scan which showed no alterations, and he was treated with diphenylhydantoin (15 mg/dL). A lumbar puncture was performed, which produced a xanthochromic liquid, containing 220 mg/dL microproteins, 67 % glucose, 117.5 mm^3^ leukocytes, 26 % monocytes, and 74 % polymorphonuclear cells. He was evaluated by pediatric neurology and cardiology and a diagnosis of suspected multisystemic inflammatory syndrome associated with COVID-19 was established. In addition, the epidemiology department gave a presumptive diagnosis of rickettsiosis, so he was started on doxycycline at a dose of 4 mg/kg/day, and molecular and serological tests were requested from the RRC to confirm the diagnosis after three days of hospital care. The diagnosis of rickettsiosis was established using the polymerase chain reaction and sequencing of the fragments of the *ompB*^8^ and the *htrA* genes (17 kDa protein),^9^ which resulted in 98 % homology to *Rickettsia ricketsii*.

During his hospital stay, generalized petechial rash was observed on his palms and soles, in addition to a circular necrotic lesion on the external malleolus of the left foot. After thirteen days of hospital stay, he was discharged due to clinical improvement, with no evidence of cerebral, cardiac, or renal sequelae.

## Discussion

In Mexico, three rickettsia species of medical importance have been documented, *Rickettsia typhi*, which causes murine or endemic typhus, with a mortality rate of less than 1 %, *Rickettsia prowazekii*, which produces epidemic typhus, and *Rickettsia rickettsii*, which is the etiological agent of Rocky Mountain Spotted Fever (RMSF), the mortality of which in Mexico ranges between 20 % and 60 %.^10^^,^^11^

In Yucatan *R. rickettsia* and *R. typhi* have been found related to clinical conditions more frequently in recent years, so it has been recommended they be considered as re-emerging diseases. Overall, the suspicion of rickettsiosis turns into a challenge for clinicians because of the nonspecific nature of the initial symptoms, and the lack of integration of epidemiological clues, so that they can be bewildered with other endemic diseases in Mexico such as dengue fever, chikungunya, zika and other exanthematous illnesses that are frequently observed in children.^12^

In the two documented cases, although initial symptoms were nonspecific and similar to that of other endemic infectious febrile illnesses in the region such as Dengue, Zika, Chikungunya, among the most common, it is noteworthy that both presented petechial exanthema on palms and soles, which is a sign of endothelial and vascular involvement, usually occurring after five days of evolution in RMSF.^13^ A tick bite was documented in one of the patients, which coincides with that reported by Buckingham who points out that only 50 % of patients report such bites. In the other patient, *R. typhi* was implicated and although it was not possible to document flea bites, infestation by these arthropods is recognized in animals such as *Didelphis spp*. and other rodents that coexist with domestic animals such as cats and dogs, favoring dispersal.^14^ It is likely that despite the clinical features observed in both patients and because of the concomitance of COVID-19, the diagnosis of rickettsiosis was not suspected upon first medical examination.

In the context of the COVID-19 pandemic, the use of inflammatory markers such as D-Dimer has proven its prognostic value.^15^ Guan and collaborators (2020) observed that 46 % of the patients evaluated had increased D-dimer, rising to 60 % in those with severe disease and 69 % in patients who required mechanical ventilation or died. D-Dimer is a global marker of activation of the coagulation and fibrinolytic systems and serves as an indirect marker of thrombotic activity. Rickettsial infection induces a generalized vasculitis leading to microhemorrhages, increased vascular permeability, and edema, as well as activation of humoral inflammatory and coagulation mechanisms. It is possible then, that D-Dimer is useful as a prognostic marker in patients with rickettsiosis.^16^ In addition, it is important to evaluate other interleukins such as IL-1 and IL-6 as prognostic factors in cabinet studies, in which IL-1B in particular has been linked to inflammasomes as well as IL-6.^17^ This data would be relevant in conjunction with laboratory studies. Inflammatory markers such as D-dimer or interleukins, along with identification of the rickettsia group involved (SFG or TG), may serve as prognostic factors, especially in hyper- endemic areas.

The COVID-19 pandemic has made some medical criteria more sensitive, which may increase the likelihood of misdiagnosing endemic diseases in tropical regions.^18^ The cases reported here were admitted with an initial diagnosis of MIS-C, which may have contributed to delayed suspicion of rickettsiosis and consequently delayed initiation of doxycycline, which in turn is associated with the risk of complications and fatal outcomes in patients with RMSF.

To establish a diagnosis of rickettsiosis, in addition to correct assessment of clinical symptoms, a systematic study of the epidemiological and environmental determinants surrounding the cases should be incorporated. It is not recommended that on the first medical examination, which usually occurs within the first 72 h, laboratory and cabinet data be sought as a guide to the suspected diagnosis. However, this data is useful as prognostic markers when the symptoms are systemic and there are complications in various organs and systems.

On the other hand, it is important that in endemic regions of rickettsiosis that morphological, metabolic and genetic identification of rickettsial agents is sought, particularly those of the Typhus Group (TG) and the Spotted Fever Group (SFG), since there are fundamental differences in their pathophysiology, clinical and laboratory expression, transmission mechanisms, reservoirs, and mortality, among others.^18^ Although the target cells in an infection by *Rickettsia* spp., are endothelial cells, producing replication at the cytoplasm level, one difference between TG and SFG is that the *Rickettsiae* of the latter can also infect the cell nucleus and develop intracellular movement and intercellular dispersion through actin polymerization proteins, a property that gives it a particular ability to cause exanthema, edema and fluid extravasation, which are highly evident in severe infections by species such as *R. rickettsii* or *R. conorii*.^19^

The first case presented an infection by a *Rickettsia* of the Typhus Group (*R. typhi*) with lethality and morbidity are low and limited complications and short hospital stay have also been documented.^20^ The second case was confirmed as RMSF, displaying complications that warranted critical care.

It is important to consider that, although the diagnosis of Rickettsiosis was not made within 72 h of the onset of the disease, treatment with IV doxycycline was started from the moment of suspicion, which may have saved the patient with RMSF from a fatal outcome. Another relevant treatment to highlight is the use of intravenous human nmmunoglobulin, used in both patients, and which is considered as a coadjuvant treatment in cases of MIS-C.^21^ Regarding its use and probable benefit in patients diagnosed with Rickettsiosis, it should be noted that recent studies have demonstrated that the use of intravenous human immunoglobulin as therapy for MIS-C is linked to a decrease in IL-1β-producing neutrophils and reduced activation, in addition to inducing rapid cell death of neutrophils. Therefore, although it is not the treatment of choice in patients diagnosed with severe manifestations of rickettsiosis, its use as an immunomodulator could provide additional support during the inflammatory process, although studies should be conducted to evaluate this assumption.

## Conclusion

MIS-C should be considered in children with elevated inflammatory markers when there is a history of COVID-19 and they also meet criteria that have already been established by international agencies, such as CDC and WHO. However, when there is diagnostic uncertainty, other diseases with similar symptoms should be investigated. In areas where exanthematous febrile diseases are endemic and diverse rickettsia species are prevalent, the fact that a patient has compatible symptoms and history of contact with domestic or wild animals, should guide clinicians to diagnostic suspicion, and empirical treatment with doxycycline should be initiated. The initiation of empirical antibiotic therapy offers a margin of probable survival in life-threatening cases of patients with *Rickettsiae* of the Spotted Fever Group.

## Author's contribution

Conceptualization: KDR, DGLS; Data curation; KDR, DLS; Formal analysis: KDR, DLS, FPM; Funding acquisition: KDR.; Investigation: KDR, DGLS, SGC; Methodology: KDR, DGLS, GAH; Project administration: KDR., DGLS, FPM; Writing-original draft: KDR, DLS, GAH; Writing-review and editing: KDR, DGLS, FPM, GAH. All authors have read and agreed to the published version of the manuscript.

## Ethical considerations

The Research Ethics Committee of the O'Horan Hospital (Merida, Yucatan, Mexico) approved the ethical statements of this work, as a goal of project CIE-010-1-14.

## Conflicts of interest

The authors declare no conflicts of interest.
